# Supplementary figures and images for: Syngas from Reforming Methane and Carbon Dioxide on Ni@M(SiO2 and CeO2)
Source: Nanomaterials (Basel). 2024 Nov 22;14(23):1877. doi: 10.3390/nano14231877 (PMC11643234; doi:10.3390/nano14231877)

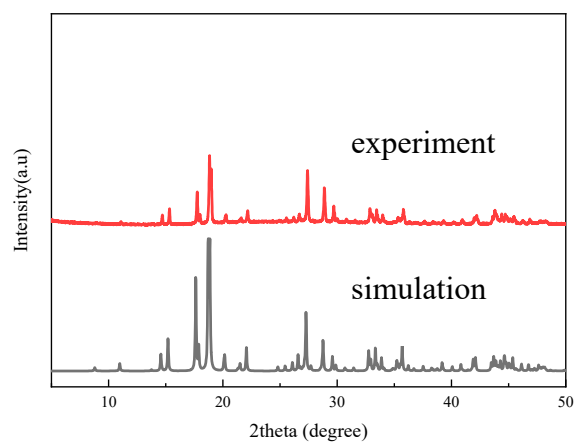

Figure S1. XRD patterns of MOFs simulation and experiment

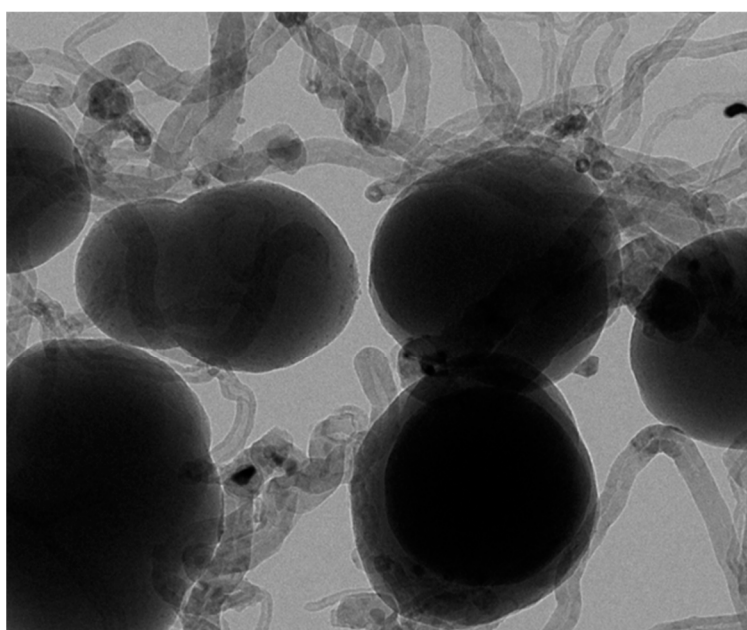

500 nm

Figure S2. Tem of Ni@CeO<sub>2</sub>(M)

Supplement: Supplementary file 1 [file nanomaterials-14-01877-s001.zip › nanomaterials-3182943-supplementary.pdf]
